# Pregnancy and Spontaneous Coronary Artery Dissection: Lessons From Survivors and Nonsurvivors

**DOI:** 10.1161/CIRCULATIONAHA.122.059635

**Published:** 2022-06-03

**Authors:** Nathan Chan, Diluka Premawardhana, Abtehale Al-Hussaini, Alice Wood, Vasiliki Bountziouka, Deevia Kotecha, Eva Swahn, Henning Palmefors, Christos Pagonis, Sofia Sederholm Lawesson, Jacek Kądziela, Marcos Garcia-Guimarães, Fernando Alfonso, Javier Escaned, Fernando Macaya, Melisa Santás, Enrico Cerrato, Angela H.E.M. Maas, Ota Hlinomaz, Nigussie Bogale, Bernardo Cortese, Mavis Cheng, Aidan Bolger, Shazia T. Hussain, Nilesh J. Samani, Marian Knight, Matthew Cauldwell, David Adlam

**Affiliations:** Department of Cardiovascular Sciences and NIHR Leicester Biomedical Research Centre, UK (N.C., D.P., A.A., A.W., V.B., D.K., M.C., A.B., S.T.H., N.J.S., D.A.).; Department of Cardiology and Department of Health, Medicine and Caring Sciences, Linköping University, Sweden (E.S., H.P., C.P., S.S.L.).; Department of Invasive Cardiology and Angiology, National Institute of Cardiology, Warsaw, Poland (J.K.).; Department of Cardiology, Hospital Universitario de La Princesa, Madrid, Spain (M.G.-G., F.A.).; Department of Cardiology, Hospital del Mar, Barcelona, Spain (M.G.-G.).; Hospital Clínico San Carlos, IdISSC, Universidad Complutense, Madrid, Spain (J.E., F.M.).; Hospital Universitario Lucus Augusti, Lugo, Spain (M.S.).; Interventional Cardiology Unit, San Luigi Gonzaga University Hospital, Orbassano, Italy (E.C.).; Rivoli Infermi Hospital, Rivoli (Turin), Italy (E.C.).; Department of Cardiology, Women’s Cardiac Health, Radboud University Medical Center, Nijmegen, The Netherlands (A.H.E.M.M.).; International Clinical Research Centre, St Anne’s University Hospital and Masaryk University, Brno, Czech Republic (O.H.).; Department of Heart Disease, Haukeland University Hospital, Bergen, Norway (N.B.).; Cardiovascular Research Team, Clinica Polispecialistica San Carlo, Milan, Italy (B.C.).; Fondazione Ricerca e Innovazione Cardiovascolare, Milan, Italy (B.C.).; National Perinatal Epidemiology Unit, Nuffield Department of Population Health, University of Oxford, UK (M.K.).; Department of Obstetrics and Maternal Medicine, St George’s Hospital, London, UK (M.C.).

**Keywords:** coronary vessels, maternal death, myocardial infarction, pregnancy

Spontaneous coronary artery dissection (SCAD) is an important cause of myocardial infarction associated with pregnancy (P-SCAD).^1–3^ It is also an understudied cause of maternal death.^4^ The present study aimed to report clinical presentation and management of P-SCAD in survivors and non-survivors and to investigate the outcome of pregnancies in women with previous SCAD.

Patients were recruited from European SCAD registries with SCAD events from 1984 to 2021. All registries were approved by national or institutional ethical review boards. All patients gave written informed consent. The P-SCAD case series consisted of 82 patients (median age, 36 years [interquartile range, 5]; 94% never/former smokers, 85% White, 15% with hypertension, 5% with diabetes, 13% with dyslipidemia, 2% with previous stroke, 22% with family history of coronary artery disease,16% with extracoronary arteriopathies, and 48% with incomplete screening). Patients were alive at the time of enrollment and presented with SCAD confirmed on invasive angiography occurring during pregnancy or within 12 months of delivery, miscarriage, or termination. The pregnancy after previous SCAD series consisted of 37 pregnancies in 28 patients with angiographically confirmed SCAD who reported a subsequent pregnancy, whether ending in live birth, miscarriage, or termination. Data on 13 patients who did not survive P-SCAD were collected from the MBRRACE-UK audit of maternal deaths.^5^

The timing of SCAD in the P-SCAD case series is shown in the Figure. Few (n = 5) SCAD events occurred during pregnancy, with the peak time of vulnerability the first month after delivery. One patient had P-SCAD 4 months after miscarriage during her first trimester. Another had P-SCAD 3 weeks after medical termination.

**Figure.**
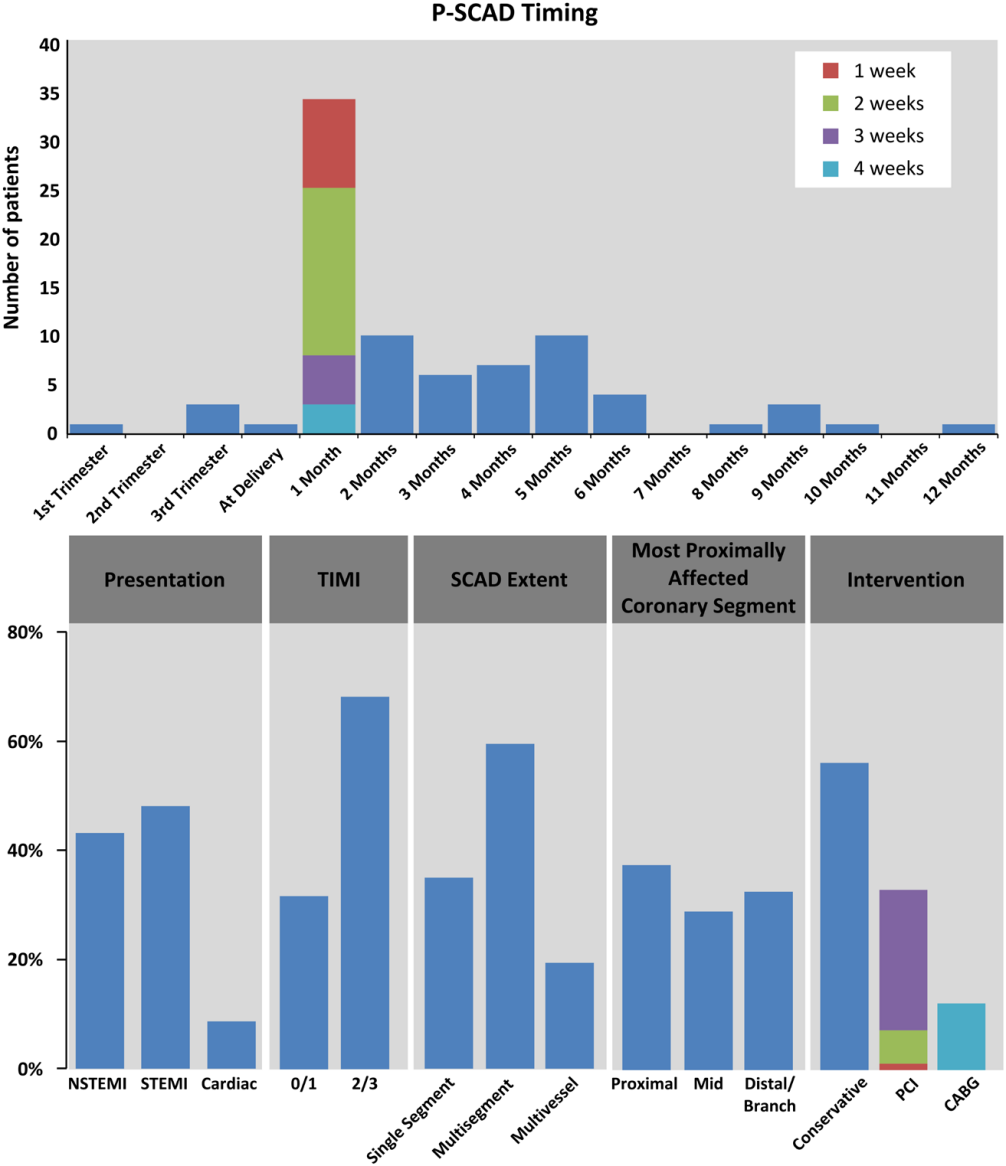
**Timing, presentation, and revascularization management of patients with spontaneous coronary artery dissection associated with pregnancy.** One patient with spontaneous coronary artery dissection associated with pregnancy (P-SCAD) did not receive angiography of the right coronary artery. CABG indicates coronary artery bypass grafting; NSTEMI, non–ST-segment–elevation myocardial infarction; PCI, percutaneous coronary intervention; SCAD, spontaneous coronary artery dissection; STEMI, ST-segment–elevation myocardial infarction; and TIMI, Thrombolysis in Myocardial Infarction.

Patients with P-SCAD had high-risk features at presentation (Figure), with 49% (40/82) presenting with ST-segment–elevation myocardial infarction. On angiography, 38% (31/82) involved proximal coronary segments, with 19% (15/81) multivessel and 57% (46/81) multisegment disease.

The proportion of patients managed conservatively with respect to revascularization was 56% (46/82; Figure). A total of 12% (10/82) were referred for coronary artery bypass surgery, and 32% (100/315) had percutaneous coronary intervention. When stents were needed, the median number of stents used was 3 (range, 1–8). The most common stented artery was the left anterior descending coronary artery. Of patients undergoing percutaneous coronary intervention, 40% (11/27) had complications during intervention (6 iatrogenic dissection, 3 hematoma extension, 1 distal occlusion, and 1 failed percutaneous coronary intervention leading to coronary artery bypass surgery).

Of the 5 women whose SCAD occurred before delivery, 2 gave birth vaginally and 3 by cesarean section (2 were planned cesarean sections because of the recent SCAD and 1 was an emergency cesarean section because of unsuccessful labor induction) with a median gestation at birth of 39 weeks (range, 31–40 weeks).

In the cohort of patients with pregnancy after SCAD, the estimated time of conception was a median of 30 months (interquartile range, 25.5) after the most recent SCAD event. Of the 28 patients who became pregnant after SCAD, in 12 cases the index SCAD event was P-SCAD. Of 37 pregnancies, 3 women opted for medical termination (in each case, because of medical advice received about the risk of pregnancy) and 7 spontaneous miscarriages occurred in 3 patients. Of women proceeding to birth, median gestation at delivery was 39 weeks (range, 36–42 weeks). A total of 41% gave birth by cesarean section. The median birthweight was at the 30th percentile, with 16% of infants small for gestational age. A total of 17 of 35 patients (49%; 2 missing data) were taking β-blockers during pregnancy. Three pregnancy-associated major adverse cardiovascular and cerebrovascular events occurred among the 37 patients (8%). One occurred during pregnancy (recurrent acute myocardial infarction at 19 weeks of gestation, likely recurrent SCAD but managed without invasive angiography) and 2 angiographically confirmed recurrent SCAD events occurred within 12 months of delivery. There were no maternal or neonatal deaths as a consequence of these events.

Of 13 maternal deaths in MBRRACE-UK attributable to SCAD, 3 occurred during pregnancy and 10 occurred postpartum (median, 16 days postpartum [range, 10–94]). Twelve women had an out-of-hospital cardiac arrest. Three women underwent angiography, including the woman presenting alive to the hospital and 2 women who had angiography during active resuscitation. None underwent revascularization. Three women are recorded as having reported symptoms before cardiac arrest. At postmortem examination, sites of coronary dissection were mostly proximal (7 of 10; 3 not reported), with the left anterior descending the most common coronary location (9 of 11; 2 not reported). One-quarter (3 of 11; 2 not reported) had multivessel dissections. Histopathologic evidence of myocardial necrosis or infarction was seen in 8 of 11 (2 not reported), with evidence of extracoronary arteriopathy reported in one.

The data, analytic methods, and study materials will be made available to other researchers on reasonable request to the corresponding author for purposes of reproducing the results. The data presented are observational. We acknowledge the potential for selection bias.

P-SCAD predominantly occurs in the first 6 months postpartum, with few cases occurring during pregnancy. Although P-SCAD has an aggressive phenotype, many women are managed conservatively with favorable outcomes. Most deaths in P-SCAD result from sudden fatal arrhythmia with little apparent opportunity for medical intervention. Pregnancy after SCAD carries a modest recurrence risk, which should be discussed as part of individualized preconception counseling.

## Article Information

### Acknowledgments

The authors thank the patients; their clinical colleagues throughout Europe; the leadership of the European Society of Cardiology–Association for Acute Cardiovascular Care spontaneous coronary artery dissection study group; Jenny Middleton, Jane Plume, Donna Alexander, Daniel Lawday, and Andrea Marshall for their support for spontaneous coronary artery dissection research; and Alberto Boi, MD, Cardiology Division, Azienda Ospedaliera Brozzu, Cagliari, Italy; Italo Porto, MD, PhD, Cardiology Division, Ospedale San Martino, Genova, Italy; and Annamaria Nicolino, Cardiology Division, Ospedale Santa Corona, Pietra Ligure, Italy, for support.

### Sources of Funding

This study was supported by the British Heart Foundation (grant PG/13/96/30608), the National Institute for Health and Care Research rare disease translational collaboration, the Leicester National Institute for Health and Care Research Biomedical Research Center, and Beat SCAD. Dr Knight is a National Institute for Health and Care Research Senior Investigator. The views expressed are those of the authors and not necessarily those of the National Health Service, the National Institute for Health and Care Research, or the Department of Health.

### Disclosures

Dr Adlam has received research funding from Abbott Vascular to support a clinical research fellowship and AstraZeneca for unrelated research and has performed consultancy for General Electric to support research funding. The other authors report no conflicts.
